# Possible factors influencing the duration of hospital stay in patients with psychiatric disorders attempting suicide by jumping

**DOI:** 10.1186/s12888-017-1267-5

**Published:** 2017-03-20

**Authors:** Tsubasa Omi, Hiroshi Ito, Keisen Riku, Koji Kanai, Hiromune Takada, Satoshi Fujimi, Hidenori Matsunaga, Kazutaka Ohi

**Affiliations:** 1 Department of Psychiatry, Osaka General Medical Center, Osaka, Japan; 2 Department of Emergency Medicine, Osaka General Medical Center, Osaka, Japan; 3 Department of Neuropsychiatry, Kanazawa Medical University, Ishikawa, Japan

**Keywords:** Suicide attempts by jumping, Schizophrenia, Hospital stay, Lower-limb fracture, Antipsychotics

## Abstract

**Background:**

Patients with psychiatric disorders have a high rate of suicide. The present study investigated factors influencing hospital stays for Japanese patients with psychiatric disorders attempting suicide by jumping.

**Methods:**

We diagnosed all suicide attempts (*n* = 113) by jumping based on the International Classification of Diseases 10th Revision (ICD-10) and investigated the mean hospital stays of patients with each diagnosis based on the ICD-10 code. We then analyzed differences in the demographic and clinical characteristics between the diagnostic groups to identify factors influencing the duration of hospital stay.

**Results:**

Patients diagnosed with schizophrenia (F2 code) were the most frequent (32.7%) of all diagnoses; therefore, we divided the diagnostic groups into schizophrenia group (*n* = 37) and other psychiatric diagnoses group (*n* = 76). The patients with schizophrenia showed a significantly longer hospital stay (125.7 ± 63.9 days) compared with the patients with other psychiatric diagnoses (83.6 ± 63.2) (*β* ± *SE* = 42.1 ± 12.7, *p* = 0.0013), whereas there was no difference in the jump height between the two groups (the average was the 3rd to 4th floor; *p* > 0.05). The number of injured parts, particularly lower-limb fractures, was significantly higher (*p* = 0.017) in patients with schizophrenia than in patients with other psychiatric diagnoses. The duration of psychiatric treatment in patients with schizophrenia were significantly longer (*z* = 3.4, *p* = 0.001) than in patients with other psychiatric diagnoses.

**Conclusion:**

Our findings indicate that the number of injuries and the body parts injured in patients with schizophrenia are associated with a longer duration of hospital stay following a suicide attempt by jumping. The current use of antipsychotics and a longer duration of taking antipsychotics might contribute to the risk of bone fracture via hyperprolactinemia. Further cognitive impairment in patients with schizophrenia might prevent rehabilitation for the management of lower-limb fractures. From these results, we suggest that clinicians should monitor the level of prolactin and cognitive function in patients with schizophrenia in future studies on managing of lower-limb fractures.

## Background

Suicide is a serious public health problem in Japan. The recent Japanese suicide prevention system has decreased the number of people who commit suicide every year since 2010. However, the problem of suicide remains and affects more than 25,000 people per year in Japan. The high rate of suicide in Japan may be caused by several reasons, including the economic recession and the rising unemployment rate [1]. Psychiatric disorders have been considered main reasons for suicide [2]. In a suicide prevention manual, the World Health Organization (WHO) reported that 90% of patients who completed suicides have psychiatric disorders [3]. Of all psychiatric disorders, the suicide risk in schizophrenia has been demonstrated in a number of studies [4, 5], and the mortality rate for suicide was found to be higher in patients with schizophrenia than in the general population. The lifetime suicide rates in patients with schizophrenia are estimated to be 4–10% [6, 7]. The risk factors for suicide in patients with schizophrenia include a history of suicide attempt, male sex, a poor adherence to treatment, a depressed mood and alcohol dependence [4, 8]. The risk factors for suicide attempt in patients with schizophrenia include depressive episodes, hopelessness and greater insight into the illness [9]. Therefore, the relationships between attempted suicide and suicide in patients with schizophrenia have been frequently reported. In contrast, few studies have reported that factors on medical treatments after suicide attempted were evaluated.

Almost all individuals who attempt suicide and are transported to an advanced emergency medical service center suffer from psychiatric disorders [10]. In a study that evaluated suicide attempts among individuals with a psychiatric disorder admitted to a hospital emergency department in an urban city in Japan, the most common method for suicide was drug overdose, followed by laceration, and then jumping from a height [11]. Fall injuries require physical treatment and rehabilitation as well as psychiatric treatment; in addition, these injuries take a long time to treat because there are often multiple injuries. However, Japanese advanced emergency medical service centers do not frequently have departments of psychiatry and rehabilitation. Therefore, some suicide attempters with psychiatric disorders are not able to receive comprehensive medical treatment. Non-comprehensive treatment in patients with fall injuries causes a long length of hospital stay, severe motility disturbances and an acute exacerbation of psychiatric symptoms. There are 768 inpatient beds at the Osaka General Medical Center (OGMC), including an advanced critical care center (18 beds), a psychiatric inpatient unit (34 beds) and an advanced rehabilitation center (87 beds). Patients with combined physical and psychiatric disorders are able to receive psychiatric and physical treatments and rehabilitation in a cooperative system in our center. Patients with fall injuries who do not have access to sufficient treatment are transferred to the OGMC to receive psychiatric treatment and rehabilitation.

We previously demonstrated that jumping from a height was significantly associated with schizophrenia spectrum disorders among suicide attempters with psychiatric diagnoses, whereas self-stabbing was significantly associated with mood disorders [12]. Little is known regarding the differences in the diagnostic and clinical characteristics in suicide attempters by jumping. The purpose of this study was therefore to evaluate factors influencing hospital stays among Japanese patients with psychiatric disorders attempting suicide by jumping.

## Method

### Subjects

We performed a retrospective study at the OGMC in Osaka, the second largest city in Japan. The subjects for this study consisted of 113 inpatients (22.1% males, 25 males/88 females, mean age ± *SD*: 37.3 ± 15.3 years) who were hospitalized in the psychiatric inpatient unit in the OGMC after they attempted suicide by jumping during the time period of January 1, 2009, to December 31, 2014. Some of the patients were transported to the trauma and critical care unit in the OGMC, where they were treated before being hospitalized in the psychiatric inpatient unit. Other patients were transferred from the psychiatric inpatient unit to the advanced rehabilitation center in the OGMC.

All patients and their relatives, if available, on admission after the suicide attempt were subjected to face-to-face interviews by at least two trained psychiatrists. A psychiatric history was obtained, and a physical examination and routine laboratory screening were performed. The diagnosis was made based on the criteria of International Classification of Diseases 10th Revision (ICD-10). Clinical diagnoses based on the ICD-10 were mainly classified as follows: ‘F0’- ‘Organic, including symptomatic, mental disorders’; ‘F1’- ‘Mental and behavioral disorders due to psychoactive substance use’; ‘F2’- ‘Schizophrenia, schizotypal and delusional disorders’; ‘F3’- ‘Mood disorders’; ‘F4’- ‘Neurotic, stress-related and somatoform disorders’; ‘F5’- ‘Behavioral syndromes associated with physiological disturbances and physical factors’; ‘F6’- ‘Disorders of adult personality and behavior,’ ‘F7’- ‘Mental retardation’; ‘F8’- ‘Disorders of psychological development’; and ‘F9’- ‘Behavioral and emotional disorders with onset usually occurring in childhood and adolescence.’ The following demographic information was assessed by the interviews: height (floor) of the suicide attempts by jumping and the clinical history of psychiatry, including age at the first visit (years), the duration of psychiatric treatment and education (years). Details of the injured body parts (head, chest, abdomen, spine, pelvis, arm or leg) were evaluated by inspection using X-ray examination, computed tomography (CT) and magnetic resonance imaging (MRI).

### Statistical analyses

All statistical analyses were performed using IBM SPSS Statistics 19.0 software (IBM Japan, Tokyo, Japan). Based on the assumption that most demographic variables, such as age and years of education, were not fitted to a normality distribution with the Kolmogorov-Smirnov test (*p* < 0.05), the differences in continuous variables, such as age and years of education, were analyzed using the non-parametric Mann-Whitney *U*-test; the differences in categorical variables, such as gender or ratio of subjects taking antipsychotics, were analyzed using Pearson’s *χ* ^*2*^ test. As mentioned in the results section, the patients were divided into two groups, a schizophrenia group and an other psychiatric diagnosis group. To identify factors influencing the duration of hospital stay between patients with schizophrenia and those with other psychiatric diagnoses, we performed multiple linear regression analyses with the duration of hospital stay as a dependent variable and diagnosis status (F2 and other than F2), height (floor) and the number of injured body parts (head, chest, abdomen, spine, pelvis, arm or leg) as independent variables. Age and gender were included as covariates to control for confounding factors. We further examined the differences in demographic and clinical characteristics between the two diagnostic groups. All *p* values were two tailed, and statistical significance was defined as *p* < 0.05.

## Results

### Psychiatric diagnosis classification due to suicide attempt by jumping

We diagnosed the 113 suicide attempts by jumping based on the ICD-10 (Table 1). The numbers (%) of each diagnosis according to ICD-10 codes are as follows: F0; *n* = 10 (8.8%), F1; *n* = 9 (8.0%), F2; *n* = 37 (32.7%), F3; *n* = 21 (18.6%), F4; *n* = 11 (9.7%), F5; *n* = 0 (0%), F6; *n* = 19 (16.8%), F7; *n* = 3 (2.7%), F8; *n* = 3 (2.7%), and F9; *n* = 0 (0%). Of these diagnostic codes, the F2 code was most frequent (32.7%). The second most common code was F3 (18.6%), and there were no individuals with F5 or F9 codes (0%).

**Table 1:** The ICD-10 classification of mental and behavioral disorders of patients with suicide attempts by jumping

ICD-10 code	Diagnostic category	No. jumping (%) *n* = 113
F0	Organic, including symptomatic, mental disorders	10 (8.8)
F1	Mental and behavioral disorders due to psychoactive substance use	9 (8.0)
F2	Schizophrenia, schizotypal and delusional disorders	37 (32.7)
F3	Mood disorders	21 (18.6)
F4	Neurotic, stress-related and somatoform disorders	11 (9.7)
F5	Behavioral syndromes associated with physiological disturbances and physical factors	0 (0)
F6	Disorders of adult personality and behavior	19 (16.8)
F7	Mental retardation	3 (2.7)
F8	Disorders of psychological development	3 (2.7)
F9	Behavioral and emotional disorders with onset usually occurring in childhood and adolescence	0 (0)

*ICD-10* International Classification of Diseases 10th Revision, Chapter V

### Mean hospital stays of each diagnosis based on the ICD-10 code

**Fig. 1:**
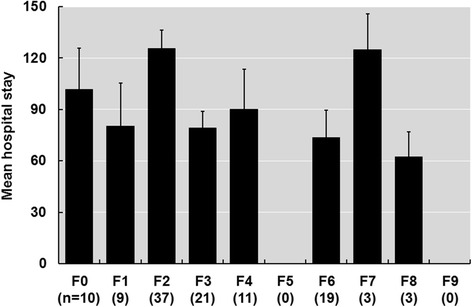
Average duration of hospital stay by diagnosis. The error bars represent the standard errors (*SE*). Patients with schizophrenia (F2) had a longer duration of hospital stay (125.7 ± 10.1) than patients with other psychiatric disorders (83.6 ± 7.4)

As shown in Fig. 1, the means ± standard deviations of hospital stays (days) in each diagnosis according to the ICD-10 code were as follows: F0; 101.7 ± 71.9, F1; 80.4 ± 70.6, F2; 125.7 ± 63.9, F3; 79.1 ± 43.5, F4; 90.2 ± 74.1, F6; 73.6 ± 67.3, F7; 125.0 ± 29.4, and F8; 62.3 ± 20.5. The mean hospital stays of patients with F2 or F7 codes were greater than 100 days. As patients with F2 codes (schizophrenia, schizotypal and delusional disorders) were more frequent in all diagnoses, we focused on patients with F2 codes for further analysis. We compared patients with F2 codes (*n* = 37) to those with codes other than F2 (*n* = 76). Of the patients with F2 codes, all patients were diagnosed with schizophrenia. The patients with schizophrenia showed a significantly longer hospital stay (125.7 ± 63.9 days) compared to the patients with other psychiatric diagnoses (83.6 ± 63.2 days) (*β* ± *SE* = 42.1 ± 12.7, *p* = 0.0013). When age and sex were included as covariates, the association remained significant (*β* ± *SE* = 40.7 ± 13.1, *p* = 0.0024).

### Influence of clinical characteristics on hospital stay

The demographic variables between patients with schizophrenia and patients with other psychiatric diagnoses are shown in Table 2. To detect possible factors influencing hospital stay, we next investigated the influence of clinical characteristics, such as diagnosis (F2 and other than F2), age, gender, height (floor) and the number of injured body parts (head, chest, abdomen, spine, pelvis, arm or leg), on hospital stay. We found that the duration of hospital stay was significantly affected by diagnosis (*β* ± *SE* = 33.0 ± 12.3, *p* = 0.0087), height (*β* ± *SE* = 10.4 ± 3.1, *p* = 0.0012) and the number of injured body parts (*β* ± *SE* = 12.7 ± 4.7, *p* = 0.0078). We further examined the differences in height and the number of injured body parts between patients with schizophrenia and patients with other psychiatric diagnoses (Table 2). The patients with schizophrenia (3.2 ± 1.2) showed a significantly higher total number of injured body parts than patients with other psychiatric diagnoses (2.6 ± 1.2) (*z* = 2.4, *p* = 0.017), whereas there was no difference in height between the two groups (*z* = −0.2, *p* = 0.83). Regarding the details of the injured parts, there were more individuals with leg injuries in the schizophrenia group (75.7%) than in the other psychiatric diagnoses group (53.9%) (*p* = 0.026). All of the leg injuries were diagnosed as lower-limb fractures. No significant differences between the two groups were found for the other injured body parts (*p* > 0.05).

**Table 2:** Demographic and clinical characteristics of suicide attempters who attempt suicide by jumping

Characteristic	F2 (*n* = 37)	Except for F2 (*n* = 76)	*p* values (*z*)
Age	41.9 ± 11.7	35.1 ± 16.3	.009 (2.6)
Gender (Male/Female)	9 / 28	16 / 60	.69 (0.2)^a^
Education (years)	12.5 ± 1.5^b^	11.6 ± 2.2	.014 (2.5)
Antipsychotic (+/−)	23 / 14	29 / 47	.016 (5.8)^a^
At the first visit (years)	29.9 ± 10.8	30.6 ± 16.3	.57 (0.6)
Duration of psychiatric treatment (years)	11.3 ± 10.7	4.5 ± 6.8	.001 (3.4)
Mean hospital stay	125.7 ± 63.9	83.6 ± 63.2	<.001 (3.7)
Height (floor)	3.6 ± 1.6	3.8 ± 1.9	.83 (−0.2)
Total numbers of injured parts	3.2 ± 1.2	2.6 ± 1.2	.017 (2.4)
Details of injured body parts			
Head (+/−)	18 / 19 (48.6%)	24 / 52 (31.6%)	.078 (3.1)^a^
Chest (+/−)	17 / 20 (46.0%)	27 / 49 (35.5%)	.29 (1.1)^a^
Abdomen (+/−)	9 / 28 (24.3%)	13 / 63 (17.1%)	.36 (0.83)^a^
Spine (+/−)	21 / 16 (56.8%)	44 / 32 (57.9%)	.91 (<0.1)^a^
Pelvis (+/−)	18 / 19 (48.6%)	28 / 48 (36.8%)	.23 (1.4)^a^
Arm (+/−)	6 / 31 (16.2%)	20 / 56 (26.3%)	.23 (1.4)^a^
Leg (+/−)	28 / 9 (75.7%)	41 / 35 (53.9%)	.026 (4.9)^a^

Mean ± *SD* are shown. ^a^ *χ* ^*2*^ test, ^b^ *n* = 36

## Discussions

To the best of our knowledge, the present study is the first to diagnose suicide attempts by jumping based on ICD-10 codes and to show the mean hospital stay for each diagnosis. The F2 code, which was applied to patients diagnosed with schizophrenia, was the most common of all diagnostic codes among suicide attempters. In addition, patients with schizophrenia showed significantly longer hospital stays compared with patients with other psychiatric diagnoses. We further investigated the clinical characteristics impacting the length of hospital stay between the schizophrenia and other psychiatric diagnoses groups. The number of injured body parts, particularly lower-limb fractures, was significantly higher in patients with schizophrenia than in patients with other psychiatric diagnoses. Nevertheless, there were no significant differences in jumping height between the two groups.

In comparison with previous reports [13–15], we show similar findings regarding the fractures in patients with schizophrenia. One possible explanation for the high risk of fractures in patients with schizophrenia is that antipsychotic-related hyperprolactinemia induces impaired metabolism of bone cells and a high rate of loss of bone mineral density [16, 17]. Kishimoto et al. [18] suggested that non-healthy lifestyle behaviors, including insufficient exercise, poor nutrition, smoking, alcohol use and low vitamin D levels, had an impact on a higher risk of fracture. A Taiwanese case-control study (605 patients and 2828 controls) found that current antipsychotic use was associated with an increased risk for hip fracture (adjusted odds ratio (OR) = 1.61; 95% CI = 1.24–2.10) and that new users had a higher risk of hip fracture (adjusted OR = 4.28; 95% CI = 1.76–10.36) compared with past users (adjusted OR = 1.11; 95% CI = 0.79–1.56) [14]. We found that the age and duration of psychiatric treatment in patients with schizophrenia were significantly older (*z* = 2.6, *p* = 0.009) and longer (*z* = 3.4, *p* = 0.001) than in patients with other psychiatric diagnoses. The majority of patients with schizophrenia had received psychiatric treatment for more than 10 years since their initial treatment. The ratio of patients taking antipsychotics was significantly higher among those with schizophrenia (62.2%) compared to those with other psychiatric diagnoses (38.2%) (*z* = 5.8, *p* = 0.016). Moreover, the dosage of antipsychotics (mg/day) was significantly higher among patients with schizophrenia (216.1 ± 303.7) compared with those with other psychiatric diagnoses (157.2 ± 435.4) (*z* = 2.7, *p* = 0.008). These findings suggest that the current use of antipsychotics and a longer duration of taking antipsychotics might contribute to the risk of bone fracture via hyperprolactinemia. However, we did not examine the level of prolactin in our patients. Thus, future studies should examine the impact of serum prolactin on managing patients with schizophrenia.

In the present study, we found that the number of lower-limb fractures was higher in the schizophrenia group compared with the other psychiatric diagnoses group (*z* = 4.9, *p* = 0.026). The management of lower-limb fractures takes a long time and requires partial weight bearing. Partial weight bearing is a common principle of postoperative treatment during the rehabilitation phase after lower-limb fracture, employing modern concepts of stable fracture fixation. Weight bearing begins on the first postoperative day and increases stepwise until full weight bearing is achieved; therefore, full weight bearing in the early postoperative phase might endanger the stability of the reconstruction and the surgical result [19].

Moreover, cognitive impairment, which is known to be a common feature in schizophrenia, might prevent rehabilitation for the treatment of fall injuries. Indeed, researchers have voiced different opinions regarding when cognitive impairments in patients with schizophrenia begin. These opinions can be broadly divided into two groups: early development, after the onset of illness, or both. Several studies have revealed that individuals who subsequently develop schizophrenia show cognitive deficits before the onset of the illness [20, 21]. Kaneko et al. [22] reported that a wide range of cognitive deficits appeared gradually after the onset of the illness and impaired patients’ functional reintegration. Although there are many studies regarding the association between cognitive deficits and antipsychotics, Husa et al. [23] and Knowles et al. [24] reported that the use of high doses of antipsychotics might be associated with decreased functions of verbal learning and memory in patients with schizophrenia after illness onset. In addition, Keefe et al. [25] reported that antipsychotic treatments improved symptoms but had little impact on cognitive function. Previous studies have frequently suggested that worsening and relapse of psychiatric symptoms may promote cognitive deficits [26, 27]. From these results, a moderate dose of antipsychotics may be sufficient to prevent suicidal attempts. Although we did not evaluate cognitive function in our samples, we suggest that future studies should examine cognitive function in relation to managing rehabilitation in patients with schizophrenia.

To date, the relationship between the risk of suicide attempt and taking antipsychotics in patients with schizophrenia has been frequently reported [7, 28, 29]. Several studies have suggested that poor adherence to medication is one of the risk factors for suicide attempts in patients with schizophrenia [7, 28]. Novick et al. [29] reported that exacerbations of psychiatric symptoms by drug deprivation could result in suicide attempts and an increased length of hospital stay. The rate of taking antipsychotics among patients with schizophrenia was low (62.2%) in the present study, which might have increased the risk of suicide attempts. The other patients with schizophrenia (37.8%) had discontinued their medications before their suicide attempts. Moreover, psychosocial factors, such as a high intelligence quotient (IQ) and a high level of premorbid function, have been associated with an increased risk of suicide in patients with schizophrenia [30]. Indeed, we observed additional years of education in patients with schizophrenia than in patients with other psychiatric diagnoses (*z* = 2.5, *p* = .014). Therefore, education may have had an impact on the risk of suicide attempts.

There might be different factors about how patients with schizophrenia fell and took a long hospital stay rather than bone density and behavior affected by cognitive impairment during fracture healing. For example, patients with schizophrenia were older and longer duration of psychiatric treatment than patients with other psychiatric diagnoses. These factors might affect the duration of hospital stay. Although we included age and gender as covariates in multiple linear regression analyses, duration of treatments was not included in our analyses. Therefore, we additionally included duration of treatments as covariates in the analysis. However, our main findings did not change even after including these confounding factors (*p* > 0.05).

This study has several limitations. First, because the participants included in this study were patients hospitalized in the psychiatric inpatient unit at a single hospital, our results were not derived from suicide attempt cases admitted to the psychiatric inpatient unit. Second, this study did not include subjects who completed their suicide attempts; in addition, some suicide attempters died before hospitalization. Third, we could not monitor the prolactin level or cognitive function in all suicide attempters, as discussed above.

Fourth, we have not evaluated the effects of psychiatric symptoms in each psychiatric disorder on length of stay. The psychiatric symptoms might lead to improper cure of physically injurious parts.

Finally, the side effects of antipsychotics, such as extrapyramidal symptoms (EPS), orthostatic hypotension and over-sedation, may have prevented the rehabilitation from treating fall injuries in this study.

## Conclusion

Our findings suggest that the presence of lower-limb fractures in patients with schizophrenia may be useful for determining the therapeutic strategy, including psychiatric and physical treatments and rehabilitation in a cooperative system. We suggest that our findings might contribute to decreasing the hospital stay of patients with psychiatric disorders attempting suicide by jumping by establishing a collaborative relationship between psychiatrists and the other doctors. However, additional hospitals that can provide both psychiatric treatment and rehabilitation are required, and further studies with larger sample sizes and more detailed clinical information are needed to assess the factors associated with a lengthened hospital stay in patients with psychiatric disorders who attempt suicide.

## Abbreviations

CI: Confidence interval
CT: Computed tomography
EPS: Extrapyramidal symptoms
ICD-10: International Classification of Diseases 10th Revision
IQ: Intelligence quotient
MRI: Magnetic resonance imaging
OGMC: Osaka General Medical Center
OR: Odds ratio
WHO: World Health Organization
